# Microfluidic Production of Multiple Emulsions

**DOI:** 10.3390/mi8030075

**Published:** 2017-03-02

**Authors:** Goran T. Vladisavljević, Ruqaya Al Nuumani, Seyed Ali Nabavi

**Affiliations:** 1 Department of Chemical Engineering, Loughborough University, Loughborough LE11 3TU, UK; G.Vladisavljevic@lboro.ac.uk (G.T.V.); R.Alnuumani@lboro.ac.uk (R.A.N.); 2 Combustion and CCS Centre, Cranfield University, Cranfield MK43 0AL, UK

**Keywords:** microfluidics, multiple emulsion, Janus drop, core/shell drops, flow focusing, microfibers, ternary drop

## Abstract

Microfluidic devices are promising tools for the production of monodispersed tuneable complex emulsions. This review highlights the advantages of microfluidics for the fabrication of emulsions and presents an overview of the microfluidic emulsification methods including two-step and single-step methods for the fabrication of high-order multiple emulsions (double, triple, quadruple and quintuple) and emulsions with multiple and/or multi-distinct inner cores. The microfluidic methods for the formation of multiple emulsion drops with ultra-thin middle phase, multi-compartment jets, and Janus and ternary drops composed of two or three distinct surface regions are also presented. Different configurations of microfluidic drop makers are covered, such as co-flow, T-junctions and flow focusing (both planar and three-dimensional (3D)). Furthermore, surface modifications of microfluidic channels and different modes of droplet generation are summarized. Non-confined microfluidic geometries used for buoyancy-driven drop generation and membrane integrated microfluidics are also discussed. The review includes parallelization and drop splitting strategies for scaling up microfluidic emulsification. The productivity of a single drop maker is typically <1 mL/h; thus, more than 1000 drop makers are needed to achieve commercially relevant droplet throughputs of >1 L/h, which requires combining drop makers into two-dimensional (2D) and 3D assemblies fed from a single set of inlet ports through a network of distribution and collection channels.

## 1. Introduction

Emulsions are dispersion of stabilised liquids within a continuous immiscible liquid, and has long been used in a verity of applications including production of pharmaceuticals [1,2,3], cosmetics [4], foods [5], electronics [6], and energy-related [7,8,9,10]. Emulsions can be fabricated as two-phase dispersions, namely simple emulsions, such as oil-in-water or water-in-oil [11,12,13] or complex multi-phase dispersions known as multiple emulsions, consisting of larger drops containing single and multiple inner compartments [14,15,16,17]. Multiple emulsions are often the intermediate products in the synthesis of microcapsules used for protecting actives from chemical degradation or evaporation [18,19,20] and for delayed/triggered release [21,22,23]. They also find numerous applications in microsensors [24,25,26], self healing materials [27,28] and material synthesis [29,30,31,32,33]. 

Conventionally, low-ordered multiple emulsions such as double emulsions are prepared by two sequential bulk emulsification steps, which include vigorous mixing to form the primary single emulsion followed by a gentle mixing to form the double emulsion [34,35,36,37]. Bulk mixing is associated with random shear distribution, and leads to the broad droplet size distribution in each step [38,39]. In addition, the applied high shear usually cause inner drops to escape from the outer drops, which results in low encapsulation efficiency [38]. However, conventional emulsification methods benefit from the large production scales which make them applicable in some industries such as food and cosmetic where the drop size uniformity is not critical [40,41]. Membrane emulsification provides an improved control over the shear resulting in the formation of double emulsions with moderate size polydispersity of 10%–20% and improved encapsulation efficiency [42,43,44,45,46,47,48]. Using this technique, however, it is not possible to control the number of inner droplets [49,50] and to encapsulate different types of inner droplets in the same outer drop [19,51,52].

Microfluidic emulsification strategies offer the fabrication of multiple emulsions with complex drop morphologies and low polydispersity of less than 5% in a highly reproducible manner [19,51,53,54,55]. They can provide 100% encapsulation efficiency and accurate and independent control of: (i) the size of the drops at each hierarchical level [54,56,57]; (ii) the number and the type of inner drops nested inside each outer drop [54,58,59]; (iii) internal structure and shape of outer drops [59]; and (iv) the rate of droplet generation [60]. Different functional ingredients can be added independently and individually into both the inner and outer drops [56] during or after drop generation. Furthermore, distinct contents of the inner droplets can also be controllably introduced in the middle phase [61].

Herein, we review recent studies on the fabrication of multiple emulsions using microfluidic devices. We first discuss different configurations of multiple emulsions and then present the microfluidic methods used for their generation. Microfluidic devices are divided into several categories and the methods used in each category are then briefly described. Finally, we discuss parallelization (scale-up) methods used for the high-throughput production of multiple emulsions.

## 2. Classification of Multiple Emulsions

**Figure 1:**
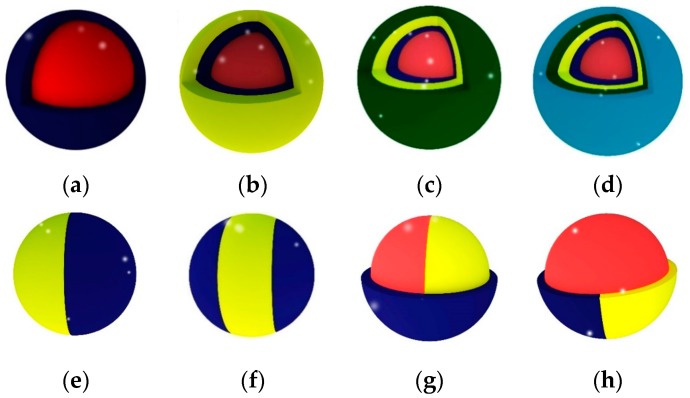

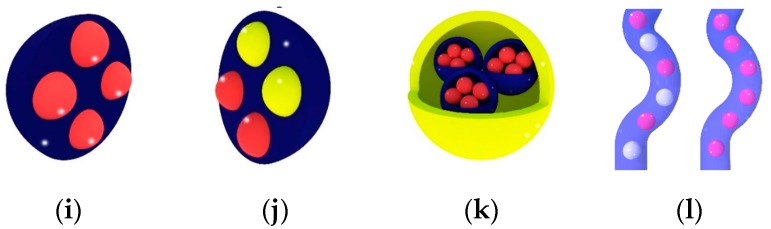
Different configurations and orders of multiple emulsion drops. (**a**) Double emulsion; (**b**) Triple emulsion; (**c**) Quadruple emulsion [15]; (**d**) Quintuple emulsion [15]; (**e**) Janus drop [64]; (**f**) Ternary drop [16,17]; (**g**) Janus core; (**h**) Janus shell [65]; (**i**) Drop with controlled number of inner drops [54]; (**j**) Drop with controlled number of distinct inner drops [66]; (**k**) High-order emulsions with controlled number of inner and middle drops [54]; (**l**) Hybrid microjet [67].

Multiple emulsion drops can be classified into four main categories: single-cored, multi-cored, Janus, and multiple-compartment. Single-cored emulsion drops are onion-like structures containing only one drop at each hierarchical level. They are composed of one core liquid and multiple concentric shells surrounding the core. Depending on the number of phases, they can be divided into double, triple, quadruple, and quintuple drops composed of two, three, four and five phases, respectively (Figure 1). Multi-cored multiple emulsion drops contain the controlled number (two or more) of inner droplets at one or more hierarchical levels. The inner drops in multi-cored emulsions can be composed of single or distinctive liquids. Janus and ternary drops are biphasic or triphasic drops having two or three physically and chemically distinct surface domains, Figure 1.

Two different types of double emulsions can be distinguished, water-in-oil-in-water (W_1_/O/W_2_) type, where two aqueous phases are separated by an oil phase, and oil-in-water-in-oil (O_1_/W/O_2_) type, where one aqueous phase separates two oil phases. Triple emulsions include W_1_/O_1_/W_2_/O_2_, O_1_/W_1_/O_2_/W_2_, O_1_/O_2_/W_2_/O_2_, etc. and can be prepared using various immiscible sets of oils [62], such as fluorocarbon and hydrocarbon oils. Moreover, water-in-water (W/W) emulsion can be generated using two immiscible aqueous solutions [63]. For example, aqueous solutions of dextran and polyethylene glycol are immiscible at high concentrations. 

## 3. Microfluidic Fabrication of Multiple Emulsions

### 3.1. Device Consideration

#### 3.1.1. Types and Geometries of Microfluidic Devices

The most common devices for microfluidic production of complex droplets and microjets are coaxial assemblies of glass capillaries [55] and planar microfluidic devices [53]. 

Planar (two-dimensional (2D)) microfluidic devices are usually fabricated by soft lithography [68], using cast-moulded stamps made from flexible materials, mainly poly(dimethylsiloxane) (PDMS) elastomer. The fabrication process starts by making a reusable master stamp, which can be used to create many replicas, by pouring over a mixture of PDMS pre-polymer, a catalyst and curing agent, allowing to cure at elevated temperature, and finally peeling off the master [68]. This method leads to the production of PDMS block with rectangular open channels, which can be sealed by thermal annealing to a glass slide or another PDMS block to produce enclosed fluid paths. Because of the rectangular shape of the channels, the continuous phase cannot completely surround the dispersed phase during the drop generation process [69]. In addition, since the inlet channel(s), through which the fluids are introduced into the device, have the same depth as the collection channel where the drops are formed, the wall of the latter can be wetted by the generated droplets which may damage the interface of forming droplets and cause failure of the drop formation [69]. Therefore, in order to controllably produce drops in planar devices, it is necessary to tune the wettability of the collection channel so that a greater affinity to the continuous phase rather than the dispersed phase is achieved and maintained. For example, a hydrophilic channel surface is required when producing O/W emulsions while the channel surface must be hydrophobic when making W/O emulsions. Consequently, to produce multiple emulsions, the channel surface is locally modified to have both hydrophilic and hydrophobic properties in different regions of the microfluidic device [52,70]. As an illustration, to generate W_1_/O_1_/W_2_/O_2_ emulsion, first W_1_/O_1_ drops are produced in a hydrophobic junction, they are then passed to the second hydrophilic junction to form W_1_/O_1_/W_2_ drops and finally, these drops are surrounded by the oil phase O_2_ in the third hydrophobic junction to produce a triple emulsion. 

For the fabrication of glass capillary devices, it is required to taper a number of round glass capillaries and align them within a square capillary or a larger round capillary [1,16,71,72]. Glass capillary devices have superior optical and chemical properties and offer straightforward surface functionalization, but are difficult to be parallelized for higher production rate of droplets.

#### 3.1.2. Surface Treatment of Microchannels for Wettability Control

● Surface modification of PDMS devices

PDMS devices can be precisely produced by soft lithography and replicated in large amounts at low cost and with complex channel configurations. However, the main drawback of inherently hydrophobic PDMS devices is the difficulty in the modification of channel wettability due to PDMS inactivity. Plasma treatment, photoreactive sol–gel coating and flow confinement methods are used to modify the wettability of PDMS channels. A common method is using oxygen plasma to oxidize the PDMS surface [73], but the surface hydrophobicity is restored after several minutes to several hours [74], which makes PDMS devices difficult to use for applications that require stable hydrophilic surface property. To get a longer hydrophilic PDMS surface, two layers are deposited on PDMS block surface by using atmospheric-pressure plasma-enhanced chemical vapour deposition (AP-PECVD). First, the surface is coated by a highly cross-linked hydrocarbon layer using CH_4_ as the reactant. Next, a hydrophilic SiO*_x_* layer is deposited using tetraethyl orthosilicate (TEOS) and oxygen. The function of the hydrocarbon layer is to act as a barrier between the PDMS and the other layer. This modification of the PDMS surface was found to be stable for 28 days [75]. 

Selective plasma treatment was used to locally modify the PDMS channel surface [73]. A scotch tape mask is placed over regions where hydrophilicity is to be retained while the rest which needs to be hydrophilic are kept open to be exposed to the ionized oxygen. An alternative method for the hydrophilic treatment of the PDMS surface is the photoreactive sol-gel coating [76]. For the photoreactive coating, a photoinitiator (Irgacure 2959) is coupled to silane (3-[triethoxysily]propyl isocyanate) and mixed with TEOS, methyltriethoxysilane (MTES), (heptadecafluoro-1,1,2,2-tetrahydrodecyl)triethoxysilane, trifluoroethanol, and aqueous HCl solution to prepare the photoreactive sol–gel mixture. To perform the coating, the channels are flushed with the photoreactive mixture and heated to 220 °C to cure the coating on the channel walls. The resulting sol-gel interface is highly hydrophobic but selective areas of the channels can be converted to hydrophilic by grafting hydrophilic patches of polyacrylic acid (PAA) onto the hydrophobic interface. To achieve this, the device was filled with a mixture of acrylic acid (AA), NaIO_4_·H_2_O, ethanol, acetone, and benzophenone and exposed selectively to ultraviolet (UV) light [76]. Alternatively, spatial control of the hydrophilic treatment was achieved by physically confining the polymerization reaction by the inert fluid and then exposing the device to UV light everywhere [77]. In this approach, an ethanolic solution of hydrophilic monomer (AA) containing Darocur 1173, a photoinitiator, is applied to the channels that need to be hydrophilic, whilst the AA solution without the photoinitiator is injected to the remaining channels.

● Surface modification of glass microfluidic devices

A significant advantage of glass capillaries is the straightforward control over their wettability by a surface reaction with an appropriate silane coupling agent [55]. For example, a highly hydrophilic glass surface can be obtained by treating the capillary with 2-[methoxy(poly-ethyleneoxy)propyl]trimethoxysilane, whilst a hydrophobic surface can be made by treating it with n-octadecyltrimethoxysilane (OTMS) [1,71,72]. The hydrophobic treatment can be achieved by dipping the capillary into 5% (*v*/*v*) OTMS solution in toluene [78] and evaporating the solvent or by exposing the capillary to OTMS vapour inside the oven at 130 °C for 15 min [79]. OTMS is deposited onto the glass surface such that the long alkyl tails form a tightly packed monolayer while the Si attaches to the substrate through Si–O covalent bonds. A plasma cleaner can also be used to render the glass surface hydrophilic [80,81].

#### 3.1.3. Droplet Formation Modes (Regimes) in Microfluidic Devices

**Figure 2:**
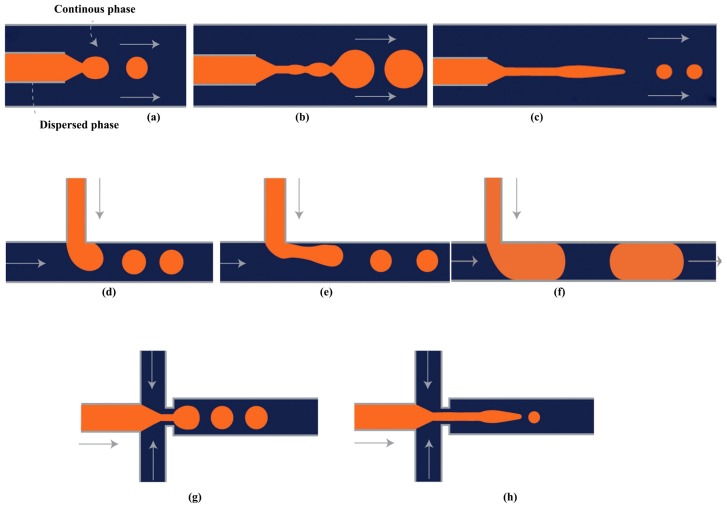
A droplet breakup in each of the three main microfluidic geometries used for droplet formation; (**a**–**c**) show dripping, widening jetting and narrowing jetting respectively in co-flow drop makers; (**d**–**f**) show dripping, jetting and squeezing in T-junction; (**g**,**h**) show dripping and jetting in flow-focusing drop maker. The orange and dark blue colors represent the dispersed phase and continuous phase respectively.

The drop formation in microfluidic devices occurs mainly in dripping and jetting regimes which are reported in all microfluidic geometries [14,82,83,84,85]. The squeezing mode is mostly reported in T-junctions and flow focusing devices [86,87,88,89,90,91,92], Figure 2. In addition, a tip streaming mode, resulting in controlled generation of submicron droplets, was reported in flow focusing devices at high viscosity ratios of the dispersed to continuous phase. The drop formation mechanism in each regime is different and governed by interaction of several forces, such as interfacial, viscous, inertial and gravitational [93]. The effect of gravity on drop formation can be neglected when the characteristic length, i.e., the drop diameter, is in the micrometre range (less than 1 mm) [94,95]. 

In the dripping regime, inertial force is negligible and interfacial force is dominant, Figure 2a,d,g. Interfacial force tends to pull back the forming drops towards the injection nozzle orifice. The drop formation begins when the viscous force exceeds the pinning force arising from the interfacial force [96] and drops are formed very close to the orifice of the injection capillary.

The jetting regime is characterised by formation of a long jet that eventually breaks into drops further downstream from the orifice, Figure 2b,c,e,h. There are two types of jetting, narrowing and widening [95]. Narrowing jetting occurs when the outer phase shearing force is dominant and considerably larger than interfacial force. Therefore, a long and thinning disperse phase jet is formed which breaks into small drops at the tip of the jet. In the widening jetting regime since the velocity of disperse phase is relatively large, the effect of inertia is significant. Once the inertial force of dispersed phase exceeds the interfacial force, a fast widening jet is developed in the collection tube and eventually ruptures to large drops. The widening shape of the inertial-driven jet is associated to the shearing force exerted by the outer phase which tends to decelerate the jet [97]. 

In squeezing mode, the shear force exerted by the continuous phase is much smaller than the interfacial force; hence, the forming dispersed phase drop keeps growing until it almost fills the entire main channel. It causes confinement of the continuous phase into a thin film between the dispersed phase drop and the wall, and results in the build-up of pressure in the continuous phase upstream of the forming drops. Due to excessive compressive force from the continuous phase, the dispersed phase neck is squeezed until it pinches off into drops [86,98,99], as shown in Figure 2f. 

In axisymmetric co-flow drop makers, the diameter of generated droplets, *d*, in dripping mode is given by [96]:

(1) $$d/D_{\mathrm{inj}}=-\delta +\sqrt{\delta^{2}+2\delta +\beta^{2}}$$

where *D*_inj_ is the diameter of injection tube, $\delta \approx \frac{60\left(Q_{d}+Q_{c}\right)\mu_{c}}{\pi D_{\mathrm{inj}}^{2}\gamma }$, and $Q_{d}$, $Q_{c}$, $\mu_{c}$, and γ are dispersed and continuous phase flow rates, continuous phase dynamic viscosity, and interfacial tension respectively. β is the ratio of collection to injection tube diameters. The size of droplets in jetting mode can be calculated using Equation (2) [83]:

(2) $$d={\left(\frac{3C^{\prime }Q_{d}}{8\pi }\frac{D_{j}\mu_{c}}{\gamma }\right)}^{1/3}$$

where $C^{\prime }$ is a function of dispersed to continuous phase viscosity ratio, and $D_{j}$ is the jet diameter. 

The size of droplets in T-junction for dripping mode can be estimated by solving Equation (3) [87,98]:

(3) $$d/d_{i}=\frac{1}{Ca_{c}}\frac{4wh-\pi d^{2}}{4wh}$$

where $Ca_{c}$ is the continuous phase capillary number, *d_i_* is cross-section dimension of the microchannel, and *w* and *h* are width and height of the microchannel respectively. The size of droplets, i.e., the length of plugs, *l*, in squeezing mode can be calculated using Equation (4) [87,98]:

(4) $$l/w=\varepsilon +\alpha Q_{d}/Q_{c}$$

where ε and α are functions of device geometry.

The size of droplets in flow-focusing drop makers can be roughly estimated from Equation (5) [99,100]:

(5) $$d/D_{\mathrm{orif}}=\;\omega {\left(Q_{d}/Q_{c}\right)}^{\epsilon }$$

where $D_{\mathrm{orif}}$ is the diameter of the orifice and, ω and ϵ are functions of the device geometry.

**Figure 3:**
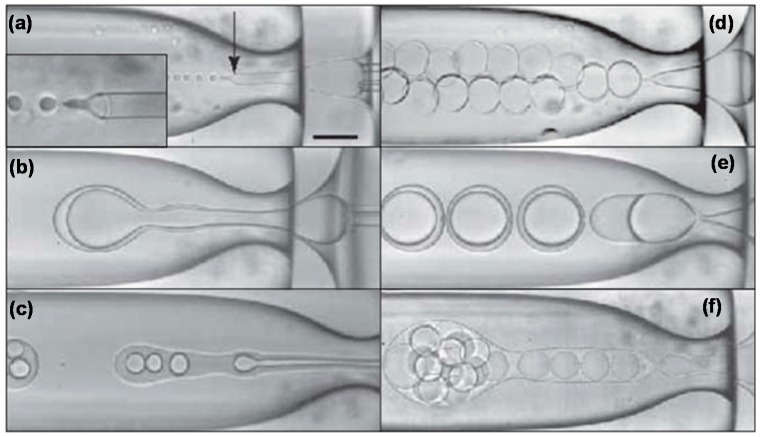
Double emulsion formation in glass capillary device in different regimes: (**a**) Narrowing jetting of inner and middle fluid; (**b**) Widening jetting of both fluids with the same break-up frequency of inner and outer drops; (**c**) Widening jetting of both fluids with different break-up frequencies of inner and outer drops; (**d**) Dripping of both fluids resulting in highly uniform core/shell drops; (**e**) Shortened jetting of both fluids. The flow pattern is similar to (**b**) but the jet length is smaller; (**f**) Extended jetting of middle fluid and dripping of inner fluid resulting in encapsulation of many inner drops in outer drops but the number of inner drops cannot be precisely controlled. The scale bar is 200 µm. Reproduced with permission from Utada et al. [14], publisher by The American Association for the Advancement of Science, 2005.

**Figure 4:**
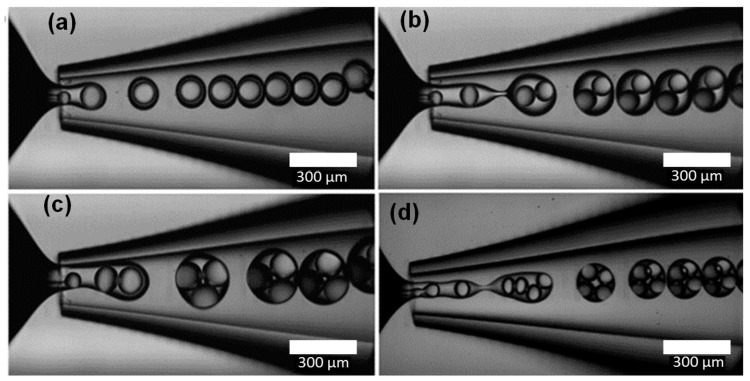
Monodispersed double emulsion with controlled number of inner drops ((**a**) One; (**b**) Two; (**c**) Three; (**d**) Four) formed in co-flow/flow focusing geometry within glass capillary device at different fluid flow rates. Reproduced with permission from Lee et al. [101], publisher by Wiley-VCH, 2009.

The same formation modes have been reported for the production of double and higher order emulsions in microfluidic devices. However, since inner and outer drops can be formed in different modes, wider variety of drop formation patterns can be achieved, Figure 3. Double emulsions are produced most controllably when both drops are formed in the dripping regime (Figure 4d), leading to very uniform drops and shells [14]. Uniform drops can also be formed in the dripping-to-jetting transitional mode. Here, the outer drops are formed in the jetting mode and the inner drops are formed in the dripping mode (Figure 4b–d). The benefit of this mode is that the number of inner drops can be controlled, typically in the range between 1 and 6 [101], by adjusting fluid flow rates, while keeping high uniformity of both inner and outer drops. 

In general, the drop formation in the jetting mode is associated with polydisperse drops, while the drops formed in the dripping mode are highly uniform in size (coefficient of variation (CV) < 3%) [55,102]. Therefore, the dripping mode is preferred in the vast majority of the applications. Typical microfluidic devices are hard to parallelize, thus, maximising the production rate while keeping the drop formation in the dripping mode is important. During the operation of microfluidic devices, the production rate can be increased by increasing the dispersed phase flow rates. However, increasing the dispersed phase flow rates is usually associated with a transition from dripping to jetting mode, and consequently production of polydispersed drops [14,83,103,104]. Therefore, it is important to supress the transition to jetting mode and maintain the dripping mode at the highest possible dispersed phase flow rates. A reduction of the shear force exerted by the continuous phase can supress the jetting mode, and can be achieved by either decreasing the continuous phase viscosity or fluid flow rates, or increasing the dimension of the main channel where the drops are formed [14,104,105,106,107,108]. In addition, an increase in the interfacial tension favours the dripping mode; thus, a reduction in concentration of the stabilisers can supress the jetting modes [102,104,105].

### 3.2. Fabrication of Double Emulsion

Double emulsion drops can be produced in single or two consecutive emulsification steps using both planar and non-planar microfluidic devices [109].

#### 3.2.1. Fabrication of Double Emulsions Using Planar Microfluidic Devices

**Figure 5:**
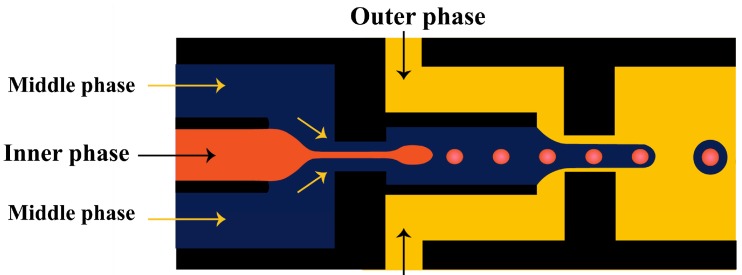
Two consecutive flow-focusing drop generators with different wettability for producing core/shell drops. The upstream and downstream orifice was wetted with the middle and outer fluid, respectively [111].

In the two-step method, double emulsions are generated in two drop makers with different surface wettabilities. The inner drops are formed in the first drop maker and then enveloped by the middle phase layer in the second drop maker. A series of two T-junctions [52,70,110], two flow-focusing units [76,111,112], two cross junctions [113,114,115], ad one T-junction combined with one flow-focusing unit [116] have been used for the two-step double emulsion production. Seo et al. [111] fabricated a PDMS device consisted of two consecutive flow-focusing drop makers with locally modified surface wettability, as shown in Figure 5. Two immiscible liquids (inner and middle phase) were injected in the first drop maker wetted by the middle phase. The outer phase was introduced in the two side inlets in the second drop maker to focus and break the middle phase jet. The core/shell drops were formed downstream of the second orifice, which was wetted by the outer fluid. The same device was used to generate core/shell drops consisting of aqueous aspirin solution in the core and aqueous solution of high molecular weight chitosan loaded with F_3_O_4_ nanoparticles in the shell [117]. The middle phase was crosslinked with glutaraldehyde to obtain magnetically responsive solid shell.

**Figure 6:**
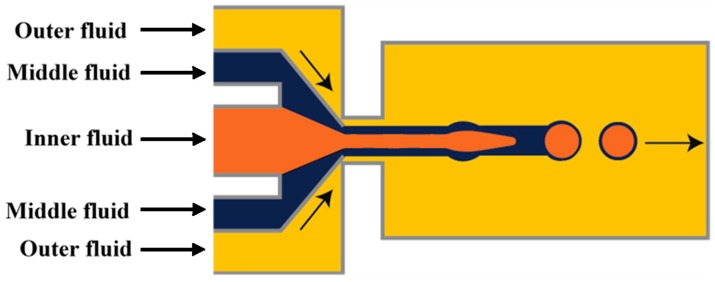
Schematic of production of double emulsion droplets in microfluidic flow focusing planar device with co-flow flow focusing geometry [118].

Nie et al. [118] fabricated core/shell drops in a single step using a double flow focusing unit fabricated in polyurethane using soft lithography, Figure 6. The aqueous continuous fluid was introduced from two side channels and the other two liquids were supplied from the central channels. A compound jet composed of the inner and the middle fluid was then focused in the orifice and the drops were formed in the downstream chamber, due to the compound jet instability. The same device was used by Zhang and co-workers [119] for the production of hydrogel capsules.

**Figure 7:**
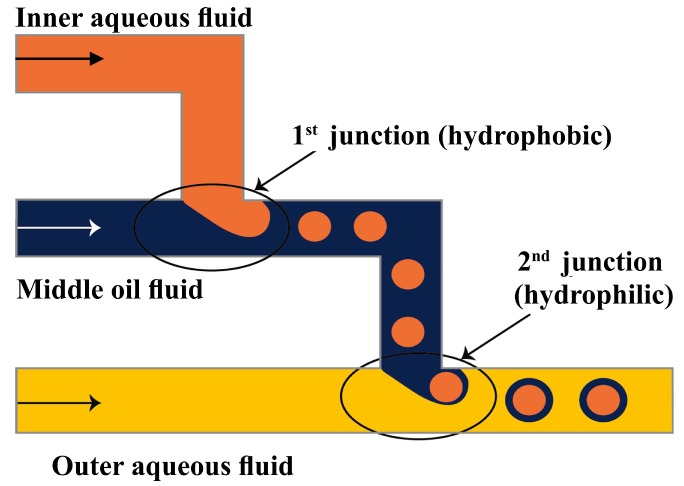
Production of W/O/W emulsions using two serial T-junctions with different wettability [70].

A Pyrex glass chip with two serial T-junctions was used for the production of double W/O/W emulsions [24,70]. The W/O drops are produced in the first hydrophobic junction followed by the formation of W/O/W drops in the second hydrophilic junction (Figure 7). The size of the drops and the number of the inner drops were controlled by varying the flow conditions and the size of the channels.

**Figure 8:**
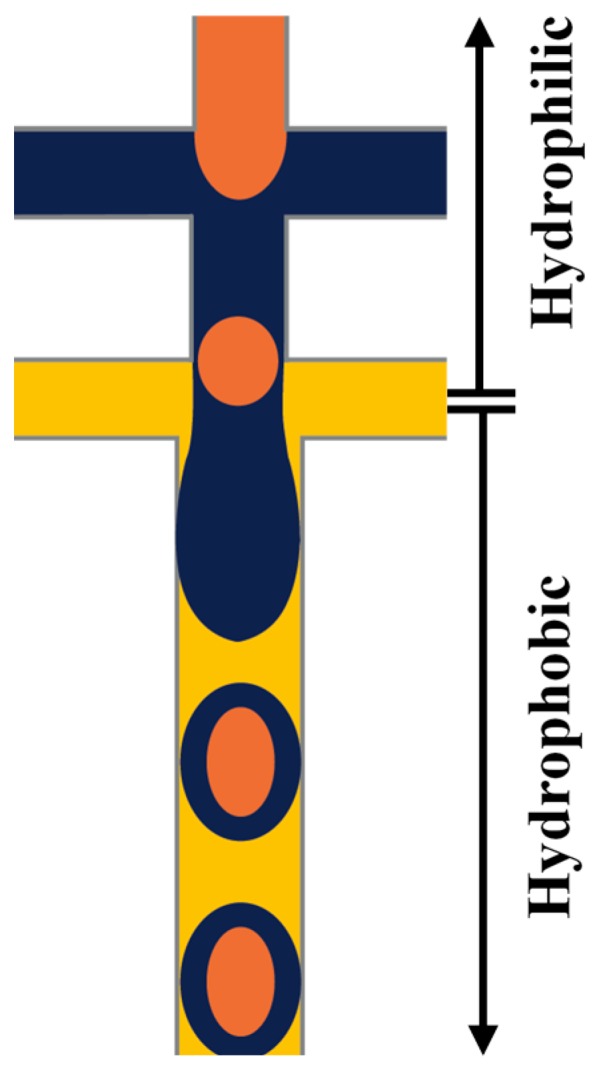
A poly(dimethylsiloxane) (PDMS) device with two serial cross junctions for formation of O/W/O double emulsion using two consecutive emulsification steps. The dripping instabilities are present in both junctions; the inner phase is emulsified at the first junction followed by the middle phase at the second junction [109].

**Figure 9:**
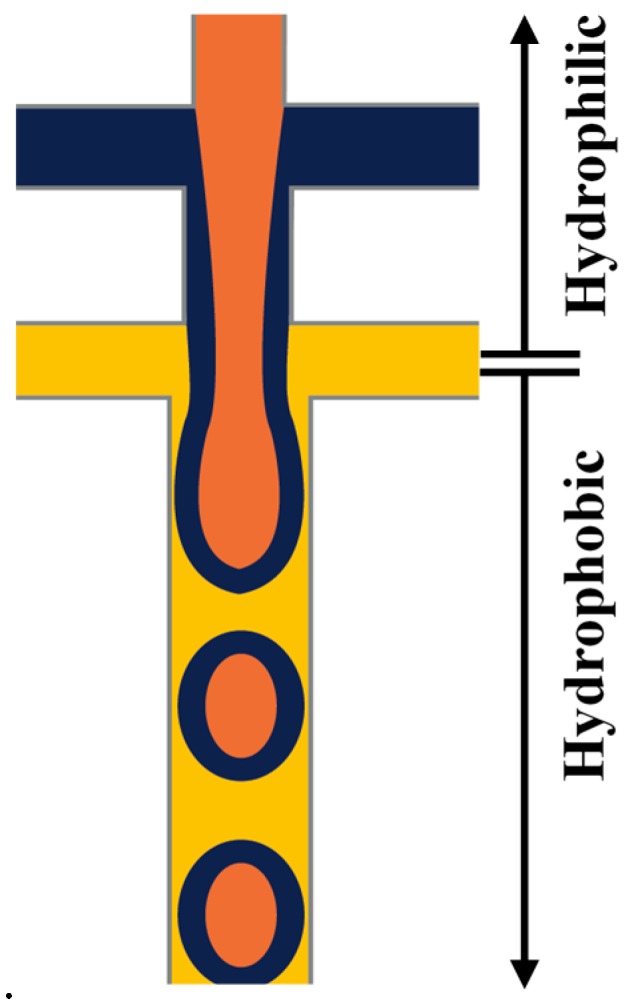
Producing O/W/O double emulsion in one step using two serial cross junctions. The first dripping instability was removed by increasing the fluid flow rates at the first junction [109].

PDMS devices with two flow focusing drop makers were used to prepare O/W/O [120,121], O/O/W [122], W/W/O [123] and W/O/W double emulsions in two emulsification steps [115,124,125]. For the formation of O/W/O emulsion, the wettability of the channels was patterned so that the first drop maker was hydrophilic and the second was hydrophobic. The inner aqueous drops were formed in the first cross junction and encapsulated within the oil drops in the second junction, as illustrated in Figure 8. This device was also used to generate double emulsions in a single step by eliminating the first dripping instability while keeping the second dripping instability, which was accomplished by increasing the flow rates in the first junction. This lead to the formation of coaxial jet of the inner and middle phase that is extended to the second junction surrounded by the outer phase which is broken to form double emulsion drops at the second junction, as shown in Figure 9. Performing one-step double emulsion formation is easier because precise patterning is not as necessary as in the two-step method since once the inner jet is formed it is surrounded by the middle fluid even when the wetting is favoured at the first junction. The generated double emulsion droplets were used as a template for the formation of polymersomes [123].

**Figure 10:**
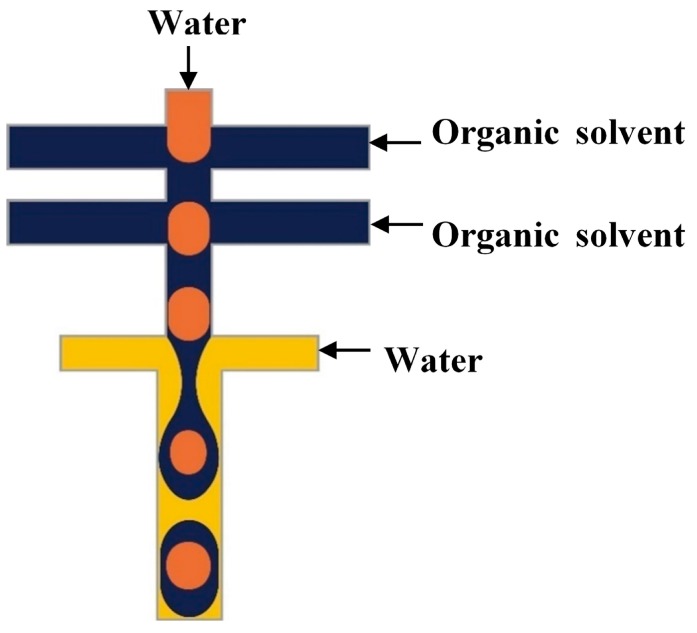
Drop maker arrays based on cross junction flow focusing geometry used to produce double emulsion droplets using two separate inlets for the middle phase [115].

Thiele et al. [115] developed a PDMS device with three sequential cross junctions coated with a solvent-resistant glasslike layer for the fabrication of polymersomes. The device has two inlets for the injection of the middle phase contents separately; one for the diblock polymer dissolved in organic solvent and the other for the second organic solvent which is miscible with the former but has different volatility (Figure 10). This configuration permitted to tune the ratio of the two organic solvents in the middle phase and manipulate the miscibility of the copolymer in the middle phase and the rate at which the solvent mixture evaporates. The separate injection of the two solvents prevented the copolymer precipitation on the channel walls that happened with premixed solvents.

#### 3.2.2. Fabrication of Double Emulsions Using Non-Planar Devices

**Figure 11:**
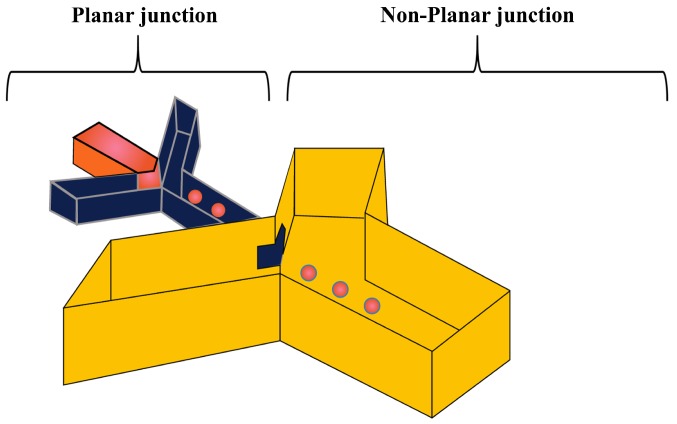
A non-planar PDMS device for making W/O/W emulsions. The inner aqueous drops are formed in the planar hydrophobic junction to be wrapped by the oil phase in the second, non-planar junction [124].

Three-dimensional (3D) microfluidic devices can minimise the contact of the forming droplets with the channel walls that normally occurs in planar devices. This is important to protect the fragile shell in the early interfacial polymerization stage from being disrupted [126] and for preventing wetting of the channels by the middle phase. A non-planar PDMS device was able to produce W/O/W double emulsions without the need for the surface modification of the channels [124]. The device was fabricated by bonding two identical PDMS moulds face to face on top of each other [127,128,129] (Figure 11). Each mould was consisted of two sequential junctions with different channel depths and the non-planar junction was formed after bonding at the transition point between the shallow and deep channel. The inner W/O droplets were generated in the planar unmodified hydrophobic junction, as the hydrophobic walls are favourable for the production of aqueous drops. They were then passed to the non-planar junction for the purpose of encapsulating the aqueous drops by the oil phase. The flow rate of the continuous phase has to be large enough in order to prevent the middle oil phase from wetting the walls of the channel in the second hydrophobic junction, but should not exceed a certain critical value limited by the bond strength of the device.

**Figure 12:**
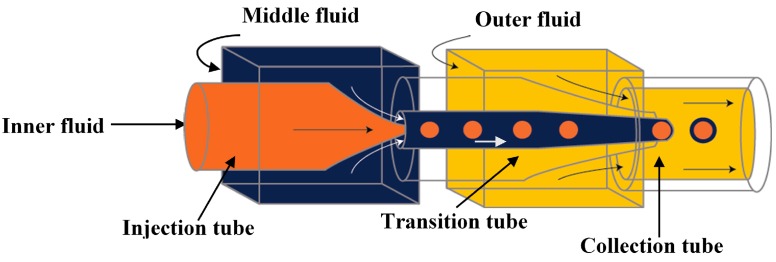
A schematic diagram of coaxially assembled glass capillaries on glass slides consisted of two co-flow drop makers for the formation of precisely controlled monodisperse double emulsions [54].

The three-dimensional (3-D) geometry of the glass capillary devices offers a controlled production of double emulsions [61,130]. These devices consist of borosilicate glass capillaries coaxially assembled on glass slides and can combine different co-flow and flow focusing drop makers [14,54,101,131,132,133,134]. A single emulsion device consists of one round capillary tube fitted inside a square capillary. In the co-flow geometry, the dispersed phase is delivered through the tapered injection tube and the continuous phase is introduced from the same direction through the coaxial area between the square capillary and the injection capillary tube [135,136]. In the flow focusing geometry, the dispersed phase is delivered through the square capillary, the continuous phase is introduced from the opposite direction through the coaxial area between the outer square capillary and the tapered inner tube, while the drops are formed and collected in the inner tube. Double emulsions were generated in two emulsification steps using two co-flow drop makers [54]. The device consisted of an injection tube (round capillary with tapered end) which was inserted in a transition tube (round capillary with a thick wall) which serves as a flow-focusing orifice for inner drops, and both were aligned within a square capillary tube. The other end of the transition tube was tapered and inserted into the collection tube (round capillary), which was also coaxially aligned (Figure 12). 

**Figure 13:**
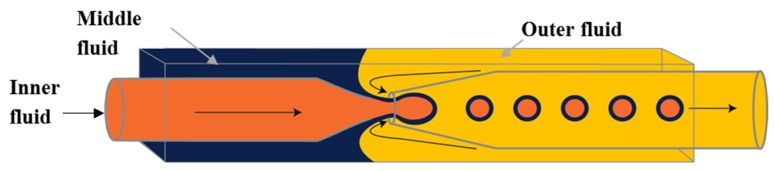
Schematic illustration of coaxial microcapillary fluidic device for making double emulsions in a single step. The geometry requires the outer fluid to be immiscible with the middle fluid and the middle fluid to be in turn immiscible with the inner fluid [14].

Utada et al. [14] has introduced a glass capillary device for the single-step generation of double emulsions. The device consisted of two rounded glass capillaries, one with hydrophilic and the other with hydrophobic tapered tips, coaxially aligned within a square capillary. The inner and the middle phase fluids were injected from the same direction through the inner round capillary and the outer coaxial region respectively, whereas, the outer carrier fluid was injected through the coaxial region in the opposite direction to flow focus the coaxially flowing fluids. The three fluids entered the orifice of the collecting tube and double emulsion droplets were formed as illustrated in Figure 13. The generated double emulsion drops can be used for encapsulation of carbon dioxide absorbents [137]. In this process, the inner phase (a CO_2_ absorbing aqueous solution) was encapsulated within the middle phase, which was the mixture of a CO_2_-permeable polymer precursor, a crosslinker and a photoinitiator, to form drops suspended in the carrier fluid. The drops were then polymerised by UV irradiation to crosslink the polymer and create solid capsules filled with the absorbent. O/W/O double emulsions are also formed using the same device with the opposite capillary surface treatment [138]. The generated double emulsion drops were also used as templates for fabrication of termoresponsive poly(N-isopropylacrylamide) microshells [139] and non-spherical giant vesicles (colloidosomes and polymersomes) with multiple compartments [101].

The size of double emulsion drops, *d*_2_, formed in dripping regime is given by [14]:

(6) $$d_{2}={\left(3\pi /2\;\mathrm{in}\right)}^{1/3}D_{j,2}$$

where $D_{j,2}$ is the diameter of the compound jet at the orifice of the collection tube, and in is the maximum instability and is a function of dispersed to continuous phase viscosity ratio. The size of double emulsion droplets in jetting mode can be estimated from [14]:

(7) $$d_{2}={\left(\frac{3C^{\prime }(Q_{i}+Q_{m})}{8\pi }\frac{D_{j,2}\mu_{o}}{\gamma_{om}}\right)}^{1/3}$$

where $Q_{i}$, $Q_{m}$, $\mu_{o}$, and $\gamma_{om}$ are the fluid flow rates of inner and middle fluid, the viscosity of outer fluid, and the interfacial tension between middle and outer fluid respectively.

**Figure 14:**
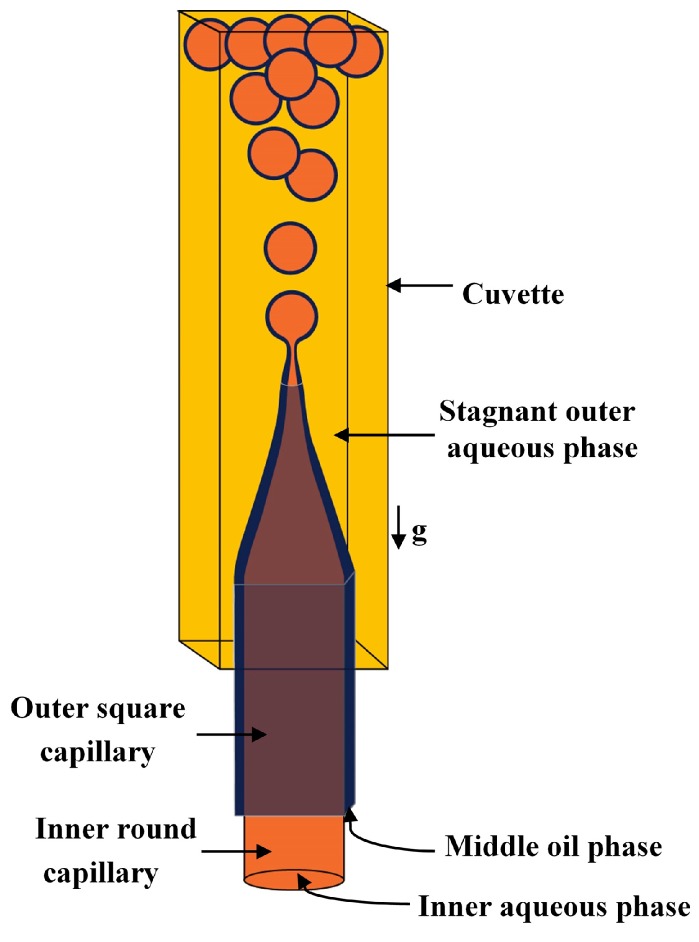
The formation of W/O/W drops with ultrathin shells in a non-confined microfluidic device consisting of coaxially aligned tapered square and round capillary tubes immersed in a quiescent outer phase [81].

Double emulsion drops with ultrathin shell can be fabricated using flow focusing geometry but the resulted drop diameters are typically 20–300 μm, due to the high drag force exerted by the continuous fluid associated with a confined geometry of the collection tube (Figure 13). Double emulsion drops with larger size (in millimetres) and ultrathin shell are favourable in some applications, particularly in biotechnological sector [140]. A biphasic flow in a non-confined system was used to generate W/O/W double emulsions with millimetre size and with ultrathin middle phase in a single step process [81,140]. Unlike in the confined system, the droplets rupture under the dominant effect of the buoyancy. The device consists of a vertically oriented outer square and inner round glass capillaries which were tapered, coupled together and aligned coaxially, as illustrated in Figure 14. Both tips had the same vertical position for fabricating core/shell drops, whereas the inner tip was kept behind the outer tip for double emulsion drops with multiple inner drops [140]. The inner surface of the outer capillary was hydrophobic to favour the flow of the oil phase in the coaxial area between the two capillaries, while the outer surface of the outer capillary was hydrophilic for the contact with the outer phase. These aligned capillaries were inserted into a cuvette which served as a non-confined reservoir for the continuous fluid. Chaurasia et al. [81] developed a phase diagram to identify the relationship between the drop geometry and the process conditions. The droplets with a radius of ~100 µm to 1 mm and a shell thickness of 2–30 µm were formed in both dripping and jetting regimes. An increase in the surfactant concentration led to an increase in the shell thickness. The ultrathin shell was achieved when the inner fluid flow rate was at least ten times the middle phase flow rate [81].

Recently, a reusable and low cost microfluidic device based on the assembly of commercial dispensing needles, mini-cross links and tree-links was introduced [141]. Double and multi-component double emulsion droplets were produced in this device ranging from micrometers to millimeters in size by adjusting the dimensions of the needles, the distance between the needles and the flow rates. The main drawback of this device is the inability to observe the emulsification process as the steel needle is not transparent.

### 3.3. Fabrication of Micron-Sized Droplets with Ultra-Thin Shell

**Figure 15:**
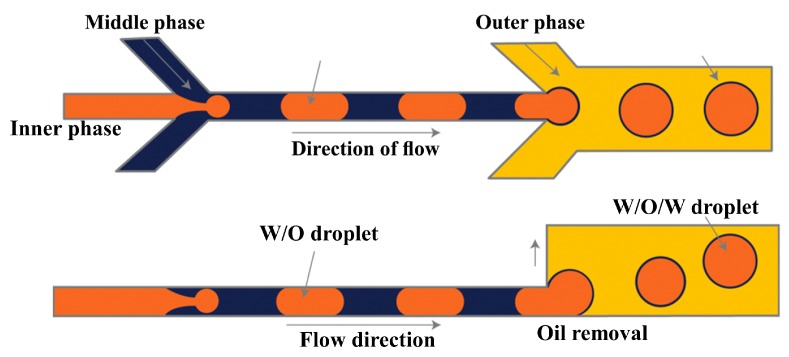
PDMS device for the formation of core/shell drops with ultra-thin shells (top and side views). The oil phase was drained from the shell by flowing away along the hydrophobic surface of the W/O/W channel [144].

Micron-sized double emulsions drops with ultra-thin shells are very useful templates for the preparation of functional vesicles and capsules with solid shells [142]. W/O/W drops with shells thinner than 1 µm were produced using a flow focusing PDMS device with two junctions with different channel depths [143,144], Figure 15. Furthermore, the device can be parallelized as it is made by soft lithography enabling high production quantities of the droplets. The second junction has a step structure and all channels are hydrophobic. The W/O drops were formed in the form of slugs in the upstream junction. The drops were then transferred to the downstream deeper junction where spherical core/shell drops were formed because the channel was deeper. Most of the oil phase was drained off from the middle phase by flowing along the hydrophobic surface of the W/O/W channel. It results in a very thin oil shell surrounding the inner aqueous phase.

**Figure 16:**
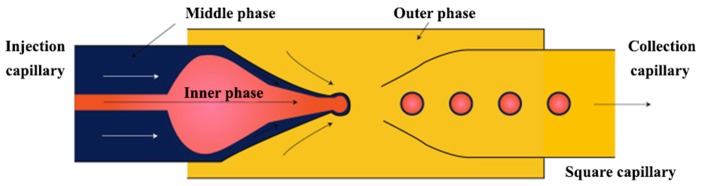
Glass capillary device for the formation of thin shelled drops. The middle phase has a higher affinity towards the wall of the outer injection capillary and flows in a thin layer along the wall of the outer capillary pushed by the inner phase injected at high flow rate through the innermost capillary [31].

Glass capillary device with two coaxial injection capillaries was used for the formation of core/shell drops with ultrathin shells [31], Figure 16. A biphasic flow was created in the outer injection capillary consisting of a thin layer of the middle phase, which has a high affinity towards the wall of the capillary and surrounds the inner phase injected through the inner injection tube. The middle phase was confined in the capillary within a thin layer due to jetting of the inner phase. The outer phase was delivered between the square capillary and the outer injection capillary. The thickness of the solidified poly(lactic) acid (PLA) shell after solvent evaporation from the middle phase was less than 100 nm.

### 3.4. Fabrication of Multi-Compartment Double Emulsions

**Figure 17:**
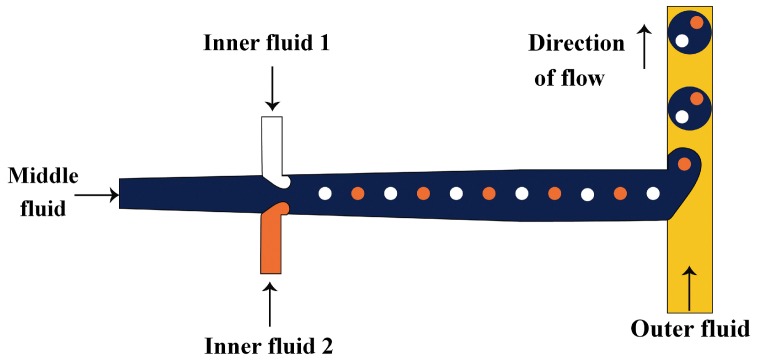
Production of double emulsion with distinct inner drops in a two-step emulsification process using the device consisted of one upstream cross junction and one downstream T-junction connected in series [70].

Microfluidic devices have been widely used for generation of double emulsion drops with distinct inner fluids [69,78,145]. This is useful for keeping mutually incompatible actives physically separated within the same microcapsule to allow synergistic effects upon their triggered release when the contents of the inner drops are combined [143]. Multiple emulsion drops with two distinct inner drops were formed by injecting different aqueous solutions in the oil phase through opposite side channels in the hydrophobic upstream junction and encapsulating two distinct cores in the same oil drop in the hydrophilic downstream T-junction [70] (Figure 17). 

**Figure 18:**
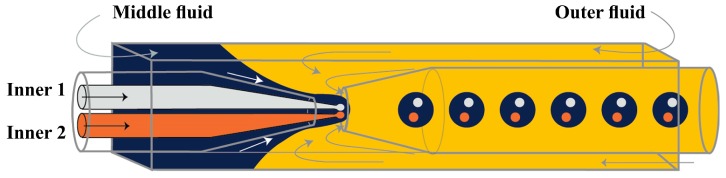
A glass capillary device with a double-bore injection capillary for preparation of multiple emulsion drops consisted of distinct inner drops using a single-step emulsification [78].

Double emulsion drops with two distinct inner cores were generated using the glass capillary device shown in Figure 18. The number and size of the inner drops was controlled by adjusting the flow rates of the three fluids. The generated double emulsions were used for the preparation of multi-compartment polymersomes [121] and solid lipid capsules [143]. Two, three and four distinct inner drops were encapsulated within the same middle phase drop by injecting the inner fluids respectively through double, triple or quadruple bore injection capillaries [78]. Double emulsions with two separate inner drops composed of quantum dots (QD)-tagged ethoxylated trimethylolpropane triacrylate (ETPTA) and ferric oxide containing ETPTA were produced using a double-bore injection capillary [146]. The inner drops were generated at the outlet of each bore and simultaneously surrounded by the outer drop. The bores were sufficiently spaced apart to prevent the coalescence of inner drops in the middle phase. The same device was used to isolate two reactants of a gas-producing reaction in two separate aqueous solutions until they are encapsulated in the same outer drop. The inner drops then merge to form gas bubbles inside the outer drops [147]. Alternatively, the inner drops are kept separated for prolonged periods of time by solidifying the middle phase in which case, the actives are released by heating the capsules above the melting point of the shell material [143]. Multiple emulsion drops with distinct inner drops can be formed by injecting a suspension composed of two different types of drops through the injection tube of the device shown in Figure 13. This method was used to produce multiple polymersomes composed of several distinct smaller polymersomes encapsulated with a single larger polymersome [148]. The orifice of the injection capillary must be larger than the polymersomes in the inner phase to prevent rupturing of their membrane during reinjection.

**Figure 19:**
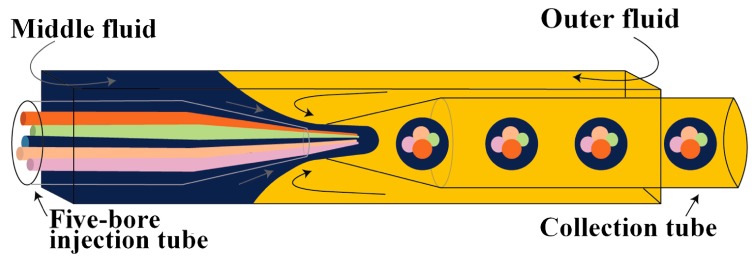
A glass capillary device for generation of W/O/W drops with four distinct inner oil drops. The injection tube has five bores, which allows four different oil phases indicated in different colours and one aqueous phase in the middle to be injected separately [66].

Zhao et al. [66] produced O/W/O emulsion with distinct oil cores using five-barrel capillary (tube with five separate internal channels) as the injection tube: four bores were used for different oil phases and one (central) bore was used to deliver the aqueous phase that was used to prevent the coalescence of the inner oil drops. The aqueous phase was also delivered through the area between the square outer capillary and the injection round capillary, as shown in Figure 19. The generated multiple emulsion drops were used as templates for the fabrication of photonic crystal barcodes composed of multiple photonic crystal or magnetically tagged cores embedded in hydrogel shell.

**Figure 20:**
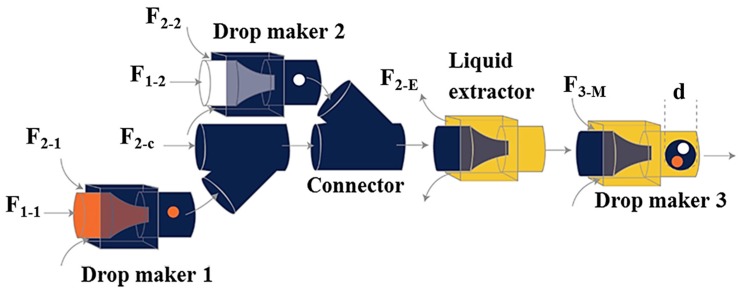
Generation of quadruple-component double emulsions using an assembly consisted of 3 drop makers, 2 connectors, and one liquid extractor. A PVC tube is used to connect the round capillaries of two adjacent drop makers. F_i-j_ is the inlet/outlet stream at each level, where the subscripts i and j refer to the level number and the injection position respectively. The drop maker 1 generates the inner drops of F_1-1_, the drop maker 2 generates the inner drops of F_1-2_, and the drop maker 3 generates the outer drops of F_3-M_ [61].

Higher order multicomponent multiple emulsions with diverse structures were produced using a flexible microfluidic device consisting of three basic building blocks: a drop maker, a connector and a liquid extractor (Figure 20) [61]. The drop maker is designed for generating drops; the connector is designed for collecting drops from different drop makers, and the liquid extractor is designed for removing excess continuous phase. This approach was used for the fabrication of capsules composed of a poly(N-isopropylacrylamide) (PNIPAM) thermo-responsive microgel shell, and oil core, and an eccentric magnetic core. The capsules were obtained by solvent evaporation and photopolymerisation of (O_1_ + O_2_)/W/O quadruple-component double emulsion [149].

**Figure 21:**
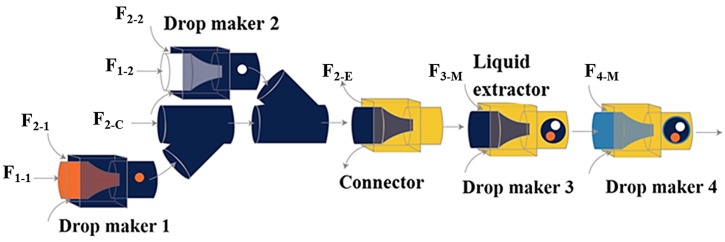
Generation of quintuple-component triple emulsions using an assembly consisted of 4 drop makers (two makers are used for the inner drops, one for the middle drops and one for the outer drops), 2 connectors, and 1 liquid extractor [61].

For fabrication of quintuple-component triple emulsions composed of five liquid phases distributed in three levels, an additional drop maker was added downstream of the main channel of the device in Figure 20 to further emulsify the quadruple-component double emulsion (Figure 21). The generated quintuple-component O/W/O/W triple emulsion consists of the red and white innermost oil droplets of F_1-1_ and F_1-2_, dispersed in an aqueous phase, which is a mixture of F_2-1_, F_2-2_, and F_2-c_ phases, surrounded by an oil layer of F_3-M_ and the whole drop is dispersed in the continuous aqueous phase (F_4-M_). The number of innermost red and white droplets can be precisely controlled, as can the number of middle drops.

**Figure 22:**
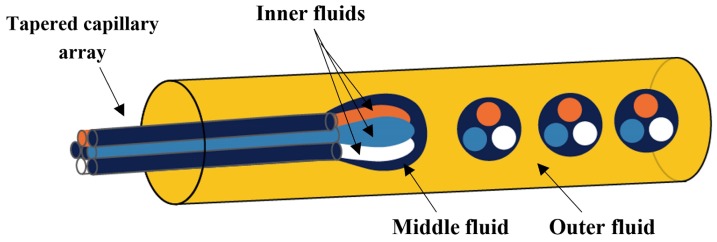
A tapered array of seven glass capillaries inserted into a collection capillary. The three distinct inner aqueous phases were delivered through nonadjacent peripheral capillaries [145].

W/O/W emulsion drops with three distinct inner drops were generated using a tapered array of seven closely packed glass capillaries inserted into a collection capillary [145]. The three distinct inner aqueous phases were injected through three nonadjacent peripheral capillaries, the middle oil phase was supplied through the remaining four capillaries, and the outer aqueous phase flowed through the interstices between the collection capillary and the injection capillary array (Figure 22). The oil phase and the outer aqueous phase were delivered into the device first. As the tapered section of the capillary array was made hydrophobic, the oil phase wetted the tip of the capillary array. Thus, when the aqueous inner phases were injected, each was in contact only with the oil phase, thus preventing coalescence of the inner phases or their release into the continuous phase. 

### 3.5. Fabrication of High-Order Multiple Emulsions

**Figure 23:**
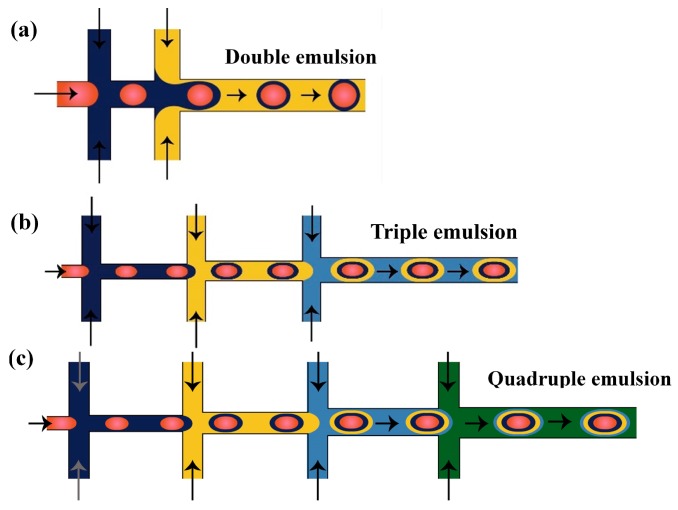
PDMS device with serial flow focusing drop makers with alternating wettability for the production of high-order emulsions: (**a**) The production of double emulsion using two drop makers; (**b**) The production of triple emulsion using three drop makers; (**c**) The production of quadruple emulsion using four drop makers [15].

Linear arrays of PDMS flow focusing drop makers with alternating wettability were used to produce droplets with multiple concentric liquids shells around the core drop [15]. The number of distinct liquids in each compound drop depends on the total number of the drop makers used. For example, to produce triple emulsions, three drop makers are used; four are used for making quadruple emulsions and five drop makers for quintuple emulsion production, as shown in Figure 23. The wettability of the drop makers depends of the type of the emulsion produced. For the production of O/W/O/W triple emulsion, the wettability of the first drop maker is hydrophilic to make the oil drops in the aqueous solution and the wettability is switched in the second drop maker to disperse the formed O/W emulsion in the oil fluid. The third drop maker is set to be hydrophilic to disperse the O/W/O emulsion in the outer aqueous phase to produce the triple emulsion. 

**Figure 24:**
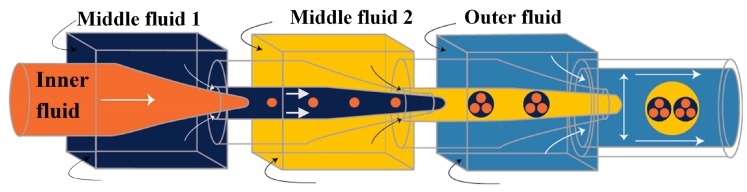
Generation of quintuple-component triple emulsions in a microfluidic device consisted of three co-flow drop makers [54].

High-order multiple emulsions have also been fabricated using sequential co-flow drop makers shown in Figure 12. Triple W/O/W/O emulsions were generated by adding a second transition tube at the outlet of the first transition tube (Figure 24) [54]. The innermost droplets are formed in the first transition tube, the middle drops in the second transition tube, and the outermost drops in the collection tube. Both transition tubes have narrow internal channels to generate small droplets. A precise control over the diameter and the number of droplets at each level was obtained. A similar flow configuration was applied to produce a coaxial jet composed of four aqueous solutions (W_1_, W_2_, W_3_ and W_4_), that served as a template for hollow Ca-alginate microfibers [150]. The core solution (W_1_) was used to create a lumen of the fiber and W_3_ was an intermediate solution used to control the rate of diffusion of Ca^2+^ from a CaCl_2_ solution (W_4_) to sodium alginate solution (W_2_), thus preventing the clogging of the capillaries resulting from rapid gelation of alginate, and enabling continuous fabrication of the fibers.

**Figure 25:**
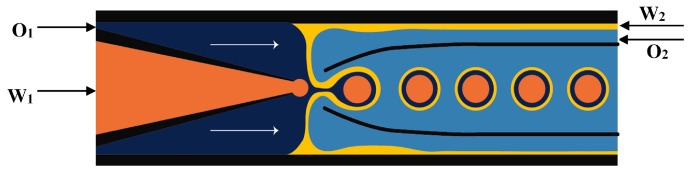
Glass capillary device for the preparation of W_1_/O_1_/W_2_/O_2_ triple emulsion consisted of one injection capillary (at the left), one collection capillary (at the right) and one small capillary (not shown here) placed between the collection and outer capillary. All mentioned capillaries are inserted in a square outer capillary. The injection and collection capillaries are hydrophobic and the square outer capillary is hydrophilic [151].

**Figure 26:**
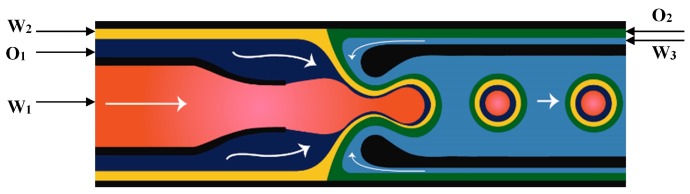
Glass capillary device for the preparation of W_1_/O_1_/W_2_/O_2_/W_3_ quadruple emulsion. One additional small capillary (not shown here) is placed between the injection and outer capillary [151].

High-order multiple emulsions can be prepared by creating stable biphasic flows in glass capillary devices with spatially patterned wettability [151]. The immiscible multiphase streams meet at the entrance of the orifice of the collection capillary and break up into complex drops with layered coaxial interfaces. For making W_1_/O_1_/W_2_/O_2_ triple emulsion, the round capillaries are modified to be hydrophobic, while the outer square capillary is modified to be hydrophilic. Moreover, a small tapered capillary is placed into the space between the collection and the square capillaries for injecting a second immiscible fluid simultaneously, as shown in Figure 25. The inner aqueous phase (W_1_) was introduced from the round injection capillary and the first oil phase (O_1_) was introduced from the coaxial region between the injection capillary and the square capillary. Both the aqueous (W_2_) and oil (O_2_) phase were injected simultaneously from the region between the collection capillary and the square capillary with the oil being introduced from the added small tapered capillary. Due to the surface treatment, the oil (O_2_) flows along the surface of the collection capillary while the aqueous phase (W_2_) flow along the wall of the square capillary. The same device was used for making quadruple emulsions but with different surface modification of the capillaries. For the production of W_1_/O_1_/W_2_/O_2_/W_3_ quadruple emulsion, both the injection capillary and the collection side of the square capillary are treated to be hydrophobic while the collection capillary and the injection side of the square capillary are set to be hydrophilic. The flows of two biphasic streams from the opposite directions of the square capillary and the inner aqueous fluid from the injection capillary are illustrated in Figure 26.

### 3.6. Fabrication of Janus Emulsion

**Figure 27:**
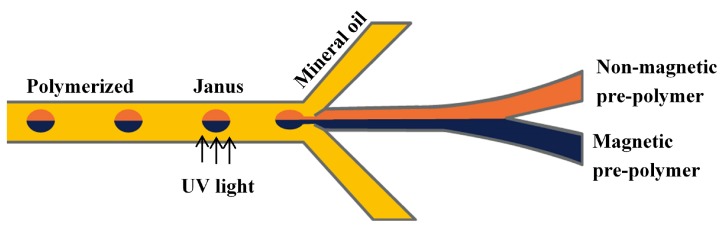
A planar microfluidic device for the fabrication of Janus particles composed of a Y-junction, a cross junction and ultraviolet (UV) lamp. Pre-polymer streams are injected from two inlets and the drops are surrounded by mineral oil at the cross junction. After UV exposure, Janus particles with magnetic anisotropy are formed [157].

Janus droplets produced in microfluidic devices are used as templates for the production of Janus particles that contain two sides with different properties [17,152,153]. For production of Janus drops from two miscible fluids in planar devices, a symmetric channel configuration is necessary in order to ensure that the two phases would not mix in the generated droplet. Typically, a Y-shaped channel was used to form a two-phase flow of two miscible dispersed phases, which then entered the sheath flow of the continuous phase in the downstream cross-junction to form biphasic Janus drops, Figure 27 [154]. An important factor is that the viscosities and flow rates of the two dispersed phases should be relatively similar to ensure a balanced width of the dispersed fluid streams and similar velocities [152]. The two dispersed phases could be a photopolymerizable phase and a non-photopolymerizable phase (silicon oil) [155], two differently coloured monomer streams containing white and black pigments [152,156], two pre-polymer solutions with and without fluorescently labelled molecules [64], and two pre-polymer streams with and without magnetite (Fe_3_O_4_) nanoparticles [157]. In situ photopolymerization of Janus drops is performed quickly, before mixing of the dispersed phase contents can take place, to preserve compositional anisotropy.

When the dispersed phases forming the Janus droplets are immiscible, e.g., organic and non-organic liquids or two immiscible organic fluids, asymmetric microfluidic devices with Y-shaped T-junction can be applied [64,157]. In the asymmetric T-junction microfluidic geometry, different flow patterns occur if the positions of the two dispersed phase streams are exchanged, leading to different morphologies of Janus drops for the same fluid flow rates [64]. 

Disk-like Janus drops with flat top and bottom surfaces and two distinct discoid compartments, rather than traditional hemispherical compartments, can be generated by adjusting the equivalent diameter of the Janus drops to be larger than the height of the main channel where the drops are formed [158]. 

The glass capillary device with a double-bore injection capillary shown in Figure 18 was used for the fabrication of double emulsion drops with the Janus core. This was achieved by switching off the middle phase flow thus allowing the change in morphology from two distinct inner drops to a single Janus drop. When the flow of the middle phase was switched on again, the middle phase formed a shell around the Janus core [146]. Janus drops were also formed in a microfluidic device composed of a pulled double-bore injection capillary inserted into the central inlet of a conventional polymethylmethacrylate (PMMA) cross junction. Two immiscible dispersed phases were delivered through separate bores of the injection capillary and the continuous phase (paraffin oil) was delivered through two side inlets of the junction [159]. When two different drops were formed at the tip of the injection capillary, they adhered together and formed a single Janus drop.

**Figure 28:**
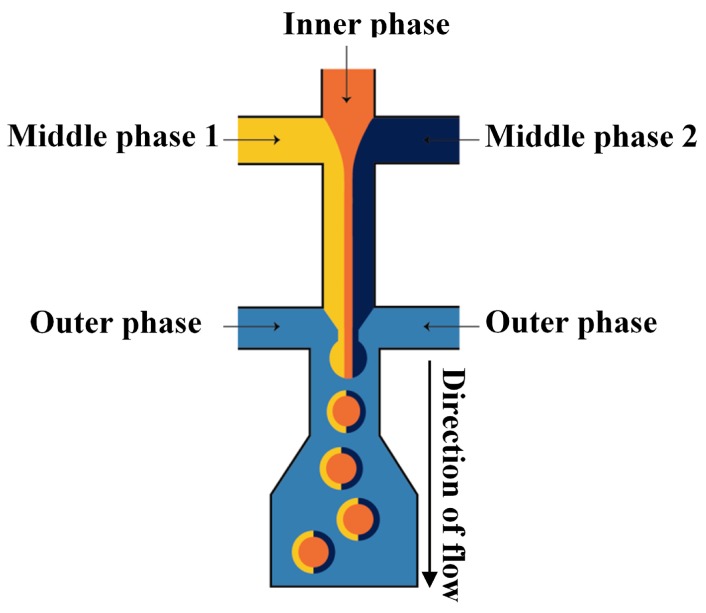
Two sequential cross junctions for generation of single drops surrounded by Janus microshells. The inner fluid forms the core of the droplets, whereas the left- and right-flowing middle fluids form a Janus-shaped shell [113].

A PDMS microfluidic device with two cross junctions was used for the fabrication of drops with the single homogeneous core drop surrounded by a Janus shell [113]. Three differently coloured solutions of poly(*N*-isopropylacrylamide) (pNIPAAm) precursor were injected through three separate inlets of the first junction, as illustrated in Figure 28. The three liquids meet in the first junction and form a laminar co-flowing stream, in which the inner phase is confined by the two distinct middle phases. Once the co-flowing stream enters the second junction, it is squeezed by the outer phase and breaks into double emulsion drops with Janus shell [113]. 

### 3.7. Practical Design Considerations

To make double emulsions (Figure 1a), microfluidic device with either single drop maker or two consecutive drop makers can be used. If single drop maker is used, double emulsion is formed in a single-step process using any drop maker consisted of three inlet (injection) channels and one outlet (collection) channel. There is no restriction regarding the channel material for the drop maker, except that the channel walls should be wetted with the continuous phase. To make W/O/W emulsion, a water-soluble surfactant such as polyvinyl alcohol (PVA) [101,115,124], sodium dodecyl sulfate (SDS) [70,81,118], Pluronic [8,61] or Tween [9,127] is added in the outer aqueous phase and an oil-soluble surfactant is added in the middle oil phase, such as phospholipids [132], di-block copolymers [115], Span [81,111], PGPR [61,149], or polyether-polysiloxane (Abil EM 90) [78,113]. The examples of such drop makers are provided in Figure 5 (flow focusing design), Figure 9 (cross-junction design) and Figure 13 (combination of co-flow and counter-current flow focusing). If two consecutive drop makers are used, double emulsion is formed in two steps: inner drops are formed in the upstream drop maker, whose walls are wetted with the middle phase and outer drops are formed in the downstream drop maker, whose walls are wetted with the outer fluid. The examples of two consecutive drop makers are provided in Figure 5 (flow focusing drop makers), Figure 7 (T-junctions), Figure 8 and Figure 11 (cross-junctions), and Figure 12 (co-flow drop makers).

For making drops with controlled number of inner drops (Figure 1i), exactly the same channel geometries can be used as discussed above, except that the fluid flow rates should be changed. The elevated inner fluid flow rate and the reduced outer fluid flow rate favours encapsulation of a larger number of inner drops [54,70,101]. 

To make triple emulsion (Figure 1b), microfluidic device with either single drop maker or three consecutive drop makers can be used. If single drop maker is used, emulsification can be accomplished in a single step using the design with four inlet channels used to deliver 4 immiscible fluids (the innermost, inner, outer, and outermost fluid) and one outlet channel used to form the emulsion. The example of such drop maker is shown in Figure 25. If three consecutive drop makers are used, formation of triple emulsion proceeds in three spatially and temporally separate steps: innermost drops are formed in the first drop maker (wetted with the inner fluid), inner drops are formed in the second drop maker (wetted with the outer fluid) and outer drops are formed in the third drop maker (wetted with the outermost fluid). The examples of three consecutive drop makers are provided in Figure 23b (cross junctions) and Figure 24 (co-flow drop makers). For making high-order emulsions with controlled number of inner and middle drops (Figure 1k), the aforementioned channel geometries can be used, except that the fluid flow rates should be re-adjusted accordingly.

To make quadruple emulsion (Figure 1c) in a single step, the drop maker should have five inlet channels to supply five immiscible fluids separately and one outlet channel to break the resulting multi-phasic stream into multi-phase drops (Figure 26). For making quadruple emulsion in multiple steps, four consecutive drop makers should be used and the emulsification takes place in four separate steps (Figure 23c).

To make quintuple emulsion (Figure 1d), to the best of our knowledge only sequential drop makers have been used [15], because it is extremely challenging to form a multi-phasic stream with six immiscible fluids that will be forced to flow through single constriction in the outlet channel.

To make Janus drops (Figure 1e), the recommended channel geometry is a combination of the upstream Y-junction to create a biphasic flow and the downstream drop maker that could be of any type used for single emulsion (Figure 2). To make (Janus core)/shell drops (Figure 1g), the downstream drop maker should be any drop maker designed for double emulsions.

To make ternary drops (Figure 1f) or (Janus core)/shell drops (Figure 1h), the recommended design is shown in Figure 28. Depending on the fluid flow rates, the resulting drop morphology will correspond to that shown in Figure 1f or Figure 1h.

### 3.8. Microfluidic Templating for Microfiber Productions

**Figure 29:**
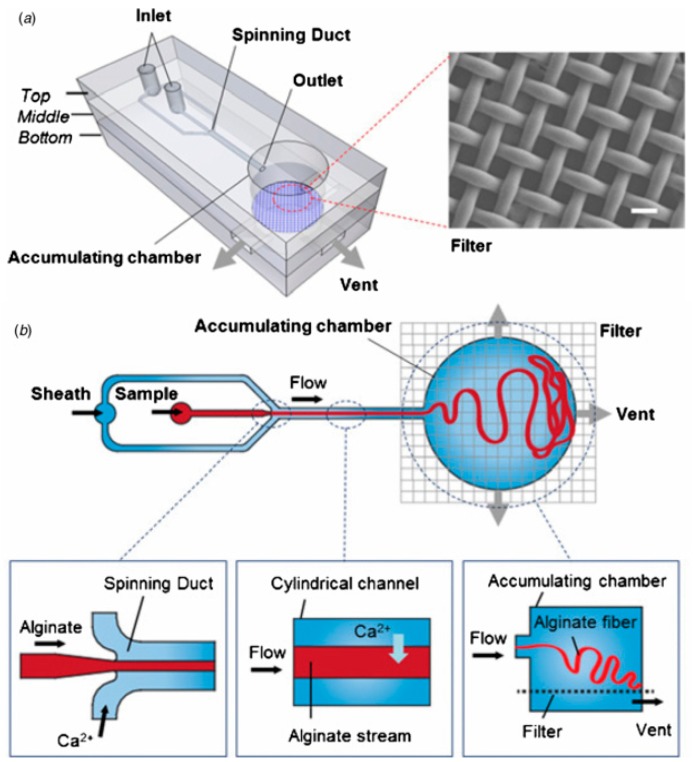
One-stop microfiber spinning and fabrication of a fibrous cell-encapsulated scaffold on a single microfluidic platform. (**a**) Schematic of the microfluidic chip used for production of fibrous scaffolds; (**b**) Schematic of the generation process of the fibrous scaffolds. Reproduced with permission from Park et al. [176], published by Institute of Physics, 2014.

Microfibers can act as high surface area micro-carriers for obtaining the controlled zero-order release profile of water soluble drugs with low efficiency of encapsulation [160,161]. Common methods used for the fabrication of microfibers are self-assembly [162], wet spinning [163], electrospinning [161,164] and electro-wet spinning [165]. Microfluidic strategies provide an easy and cheap alternative for making uniform microfibers with higher tensile strength than other methods [166,167]. Planar flow focusing devices [168,169,170] and glass capillary devices [170,171,172,173] have been used for the production of microfibers, such as alginate microfibers for the controlled release of drug in response to magnetic field and for cell culture [169] (Figure 29). The diameter of the fibers typicaly ranges from 200 to 400 μm depending on the fluid flow rates and channel dimensions. The release kinetics of the encapsulated drug from the magnetic iron oxide-loaded microfibers could be controlled externally by applying a magnetic force. In addition, these microfibers provided an environment for the growth of cells making them suitable for screening of anti-cancer drugs [169]. Collagen microfibers were fabricated using a PDMS microfluidic chip with a cross junction [174]. These microfibers can be used for peripheral nerve repair to bridge long distance gaps. A non-confined glass capillary microfluidic device was used to fabricate asymmetric oil encapsulated alginate microfibers which can be used in applications where symmetric fibers performance fail [67]. Janus microfibers with different number of hallow channels were fabricated by generating multiple laminar flows using multi-bore capillary microfluidics [175].

## 4. Droplet Splitting

Microfluidic droplet splitting strategies have been used to reproducibly generate small drops out of large drops. 3D glass capillary microfluidic device was used for the splitting of double emulsion droplets into stable multiple portions of droplets [177]. The device is composed of a droplet maker, a compression capillary, and a split capillary with segmentation obstacle. The role of the compression capillary is to deform the generated spherical droplets to plug-like shape to ensure they are aligned with the split capillary. After splitting, the resulted droplets had very narrow size distribution.

**Figure 30:**
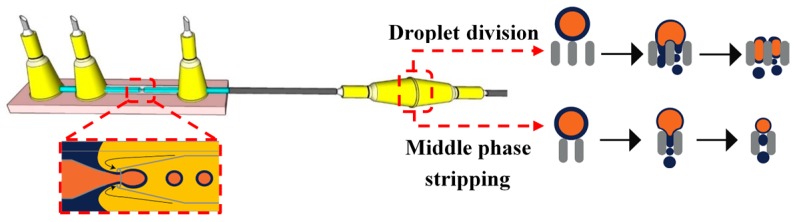
A membrane-integrated glass capillary device for the formation of small double emulsion droplets. The membrane is placed between the two Luer–Stub adapters [178].

A Membrane-Integrated Glass Capillary Device was used for the production of small-sized W/O/W emulsion drops, as shown in Figure 30 [178]. For the device fabrication, a flat SPG membrane was sandwiched between two Luer–Stub adapters and was connected with a glass capillary device using polyethylene tubing. The double emulsion droplets were generated by co-flow/flow focusing, as illustrated Figure 13. The double emulsion droplets then passed through the membrane which led to two phenomena, the division of the emulsion droplets to two or smaller droplets and the stripping of the middle oil phase resulting in thinner drop shells. 

## 5. Electric Control of Droplet Generation

**Figure 31:**
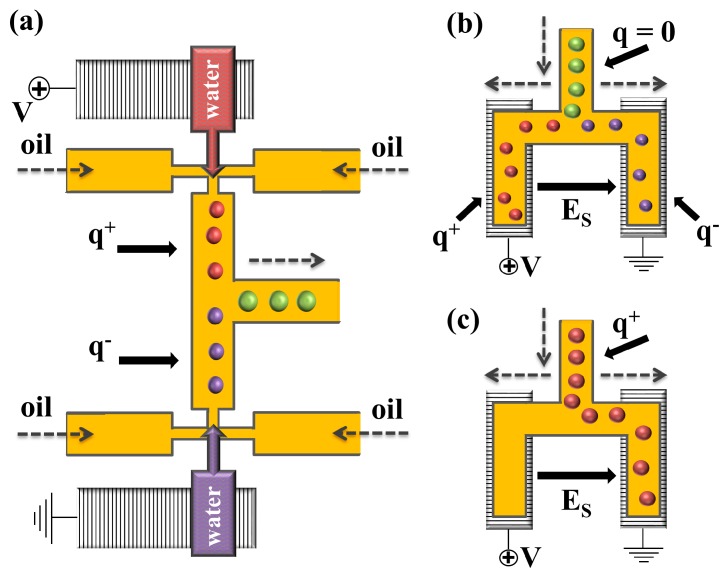
Electric control of charged droplets in microfluidic channels: (**a**) coalescence; (**b**) splitting control; (**c**) sorting control [179].

Electric control of droplets in microfluidic channels is a robust approach that can be used to actively control droplet splitting [179,180,181,182,183,184], merging [179,184,185,186,187,188,189,190,191], and sorting [179,192], which are of great interest in droplet-reaction systems, high-throughput screening, and combinatorial chemistry. In this approach, the electric field is introduced from embedded electrodes to the interface. In order to maximise the controllability of the electric field, it is necessary to place the electrode as close as possible to the domain, where the droplets are actuated. PDMS devices offer ease of structuring and have been mainly used for microfluidic electronic control. Link et al. [179] investigated splitting, coalescing, and sorting of water droplets in oil by electrostatic manipulation of droplets using embedded indium tin oxide (ITO) electrodes within PDMS channels (Figure 31a). To control the coalescence of droplets, a voltage was applied across the two aqueous streams of different compositions prior to drop generation, in order to induce opposite electrostatic charges on the generated droplets. The droplets stabilized with Span 80 (sorbitan monooleate) were carried with the continuous phase to the confluence of the two streams. The electrodes used to charge the droplets upon formation also provided the electric field to force the droplets to come together, leading to coalesce. No coalescence occurred in the absence of the electric field. The splitting of neutral droplets was achieved by polarizing them in the presence of an electric field, while entering a bifurcation, where they were split into two oppositely charged droplets, Figure 31b. The droplet sorting was accomplished by directing charged droplets to the desired channels by varying the direction of the electric field, Figure 31c. The main advantage of electro sorting is attributed to the separation of droplets based on their content instead of their size.

Mastrobattista et al. [193] used electric field to achieve dielectrophoretic separation of active and non-active enzymes encapsulated within the core of double emulsion droplets. Sciambi and Abate [183] applied an electric field across a PDMS constriction to extract the innermost fluid of W_1_/O_1_/W_2_/O_2_ triple emulsions and convert them to O_1_/W_2_/O_2_ double emulsion droplets. The electrically triggered coalescence of two aqueous cores within W_1_/O_1_/W_2_ double emulsions were reported by Jia et al. [189]. They observed that the optimized coalescing frequency increased with the medium conductivity. Electrically triggered core-coalescence of double emulsions can be used as a platform for active control of chemical and biological reactions and can be used to isolate the resultant products from the environment [189]. 

## 6. Upscaling Droplet Production in Microfluidic Devices

The major drawback of microfluidic devices is the low rate of droplet production, typically less than 1 mL/h, limiting their industrial application. The scale up the drop production, many drop makers should be joined together with the least possible number of pumps/pressure vessels and a suitable network of distribution and collection channels [194]. There are several studies reporting the operation of scaled-up systems [93,195,196,197,198]. Possible scaling-up approaches are parallelization of drop makers [199,200] and/or drop splitting [201,202,203,204]. The first approach enables to integrate a large number of drop makers onto the same chip and feed all generators from a single set of fluid inlets, i.e., only one inlet port is used to deliver one type of fluid to all drop makers [203]. Due to the large number of drop makers (>1000) needed to achieve commercially relevant flow rates (>1 L/h), 3D networks of distribution and collection channels and modular chip designs are used to implement this parallelization. The most common geometries of distribution channel network are ladder-like [200,205] and tree-like branched geometry [206,207]. The tree-like geometry has an advantage over the ladder-like geometry in providing a symmetrical distribution of the fluids and has the ability of increasing the number of the droplet makers without taking into account the hydrodynamic resistances in the network. However, for the same number of drop makers the tree-like channel network needs more space compared to the ladder-like design. For instance, the chip containing eight cross junctions connected in a ladder-like fashion requires 7 cm^2^, while the chip with the same number of junctions fed through the tree-like network is much less compact and requires 84 cm^2^ [195]. Furthermore, the tree structure is more affected by the variations in the size of the channels where a defect in one inlet or outlet will break the symmetry of the whole branch [195].

Romanowsky et al. [60] fabricated a parallelized system incorporating up to 15 double drop makers in a 2D or 3D array network, capable of producing over 1 kg per day of core/shell drops using one-step emulsification method and with droplet size variation less than 6%. Each drop maker has two sequential flow focusing cross junctions. Several drop makers are arranged horizontally and then several planar arrays of drop makers are joined vertically with a ladder-like network of distribution channels to obtain equal flow rates in all drop makers. 

Nisisako and Torii [199] have developed a microfluidic module consisting of a glass chip with parallelized drop makers fabricated by deep reactive ion etching and a multi-layer stainless steel holder with inlet channels. The fluids from vertical, radially arranged, inlet channels are supplied to individual drop makers through coaxial annular channels fabricated on the chip surface [197]. The produced droplets were collected from a common drainage port placed in the centre of the module. The module was used for the production of Janus drops/particles, and single, double, and triple emulsions. The channel geometry differs depending on the type of emulsion produced.

**Figure 32:**
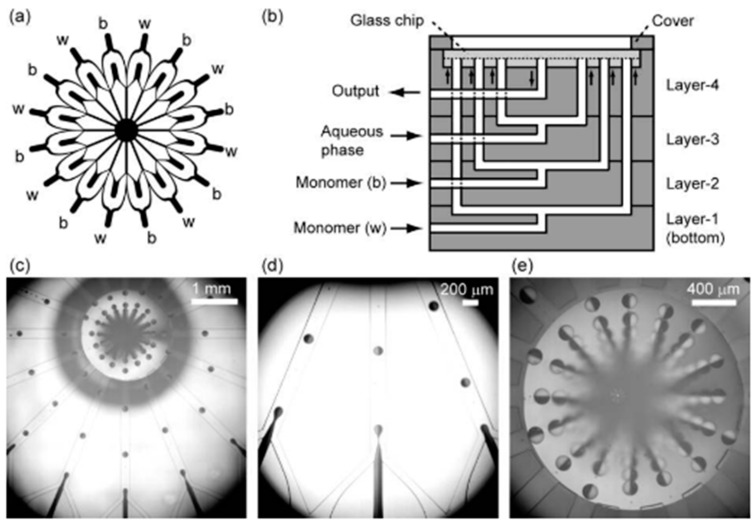
Production of Janus drops in the chip consisted of 16 Y-shaped inlet channels followed by 16 cross junctions: (**a**) Schematic of the channel geometry on the chip. The “b” and “w” labels specify the inlet positions for the black and white monomers respectively. The aqueous phase is injected from the inner 16 inlets, arranged radially with an outlet in the centre; (**b**) Schematic of the layered internal structure of the device (side view); (**c**) A micrograph showing the formation of black and white monomer drops in the module; (**d**) The magnified image of the drop makers; (**e**) Magnified image of the large outlet channel in the centre of the chip. Reproduced with permission from Nisisako et al. [152], published by American Institute of Physics, 2006.

Figure 32 shows a module with 16 drop makers used to produce 16 mL per hour of bicolored (black and white) Janus drops [152]. Each drop maker has a Y-shaped inlet junction for creation of biphasic flow followed by a cross junction for drop generation. The same module design, but with 128 drop makers, was used to generate 128 mL per hour of Janus drops with a mean diameter of ~142 μm and a CV of 3.3%. In addition, a 4 cm × 4 cm chip with 128 cross junctions was used to produce 320 mL per hour of single emulsion with a mean drop diameter of ~96 μm and a CV of 1.3%. Each cross junction had 2 drop makers, meaning that overall 256 drop makers were implemented.

Small multiple emulsion droplets were generated at a faster rate by splitting large multiple emulsion drops into two identical drops which were further divided in a series of even smaller drops using Y-shaped channels [201]. The number of the generated drops was given by 2*^N^*, where *N* is the number of sequential splitting. For example, 16 identical small droplets were obtained when one large drop was split four times. Asymmetric drop breakage was achieved by adding lateral flow at the location of a bifurcation to the device [202]. Controlling the volume (size) ratio of the produced droplets was achieved by controlling the flow rate ratio of the main and the lateral flow, which allowed the production of two daughter drops with a volume ratio up to 200:1.

## 7. Conclusions

The paper provides an overview of the recent advances in microfluidic production of conventional and complex multiple emulsions and microfibers. Multiple emulsion drops were classified in four main groups, namely, single-cored, multi-cored, Janus, and multi-compartment and the fabrication methods for each emulsion type were reviewed. Microfluidic methods are efficient tools for fabrication of highly uniform complex multi-component and multi-phase emulsion droplets with tunable morphology. The main emphasis was put on generation of multiple emulsions in planar flow focusing and T-junction drop generators and 3D glass capillary and PDMS devices, but wettability control strategies are also discussed for both PDMS and glass channels. Non-confined buoyancy driven devices enabling production of multiple emulsion drops with a diameter of up to tens of millimetres were also covered. 

Direct production of small micron-sized multiple emulsions is complicated due to wetting and fouling problems and thus, various downstream splitting strategies have been introduced to break down the generated multiple emulsions to smaller size with an added benefit of increasing the throughput. Although microfluidic chips with a single drop generation unit benefit from high controllability, they are limited to a very low production rate, and cannot be used for large-scale production. Several upscaling strategies for production of multiple emulsions have been developed, that enable increasing the production rate many times. Although these advances may satisfy small commercial applications, a suitable scalable approach for industrial-scale production is yet to be developed, and is necessary in order to further expand the applicability of microfluidic emulsification techniques.
